# Artificial Rupture of the Amniotic Sac during Labour

**Published:** 1858-01

**Authors:** B. F. Richardson

**Affiliations:** Adjunct Professor of Obstetrics and Diseases of Women and Children, in the Medical College of Ohio


*Artificial Rupture of the Amniotic Sac during Labour. Objections to the Practice* By B. F. Richardson, M. D., Adjunct Professor of Obstetrics and Diseases of Women and Children, in the Medical College of Ohio.

In the last number of this Journal, quotations were made from numerous standard obstetrical authorities, which, directly and by fair implication embody the doctrine, that the amniotic sac is the only proper and efficient agent in the production of diltation of the os uteri ; that after it is well dilated, or dilatable, there remain no important objections to rupturing the membranes ; and that, therefore, it is undesirable to maintain the integrity of the amniotic sac beyond that period. At the conclusion of that article we promised to show, "that one of its most important uses remains unfulfilled until the presenting part has passed through the superior strait ; and that in all natural, uncomplicated labors, especially primpara, the membranes should not be ruptured at any time."

If this proposition can be sustained by argument and numerous facts, the inference is patent, that the views entertained and inculcated by obstetrical writers generally, in regard to the office and treatment of the amniotic sac, are well calculated to seriously mislead those who exercise their memory more than their reason. He who conceives of no objection to the rupturing ot the membranes after the os uteri is well dilated, will, almost certainly, rupture them, in expectation of exciting more vigorous uterine contractions.

Perhaps, on no other point connected with obstetrics has there been so much misinterpretation of facts, assumptive reasoning, and erroneous conclusions, on the part of writers, as in regard to the mechanism and phenomena of the first stage of labor. As the views entertained by Professor Murphy on this point are in accordance with those of most writers, and he has elaborated the argument through which these views have generally been derived ; a review of his positions will be appropriate, in the course of my remarks.

Is the amniotic sac the only proper and efficient agent in the production of dilatation of the os uteri ?

Are there any important objections to rupturing the membranes after complete dilitation of the os uteri has taken place ?

The discussion of these questions will embody the answer to the first : Is it desirable to maintain the integrity of the amniotic sac during labor ?

Prof. Dewees, by an ingenious argument, attempts to show that, ordinarily, the amniotic sac has nothing to do in the production of dilatation ; but unfortunately he admits, in part, that which he undertakes to disprove; and moreover is guilty of palpable inconsistency. At the conclusion of paragraph 515 he writes: "but in this admission, let it be recollected that I consider the waters as useful by their equal pressure upon the lower part of the uterus, and by distending, and at the same time, by the same agency, weakening the circular fibres of the part, and thus indirectly favoring the dilatation of the mouth of the uterus." Now turn to paragraph 518. "When the os uteri does dilate, it is not by edges being stretched mechanically; it is an absolute inability in the circular fibres to maintain a state of contraction, and for the time being, may be considered as paralyzed, or excessively fatigued; or, perhaps, more properly speaking, it is the relaxation of a sphincter not subject to the control of the will." Where a proposition is faulty, the reasoning is apt to be illogical. This gifted and lamented author was endowed with a spirit of controversy to an extent that sometimes led him into the adoption and support of views, which his acknowledged powers of argumentation were insufficient to sustain. However, in attempting to exclude entirely, the amniotic sac as a usual participant in the dilatation of the os uteri, he assumed a task perhaps not more difficult than that of Prof. Murphy, who undertakes to demonstrate that it is the only proper and efficient dilating agent.

We have heretofore stated that the argument and assumption of Prof. Murphy involve "a total disregard of well known hydrostatic and mechanical laws: and that he takes the position "that the amniotic sac is a better dilater of the os, than the smooth, round and comparatively unyielding vertex." At page 55, commenting upon the argument of Dewees, he remarks: "It would make the membranes not only useless as a means of dilatation, but rather a difficulty in its accomplishment. The head of the child, directly applied to the cervix, would overcome the resistance of the circular fibres much more efficiently than the liquor amnii, so that the most favorable kind of dilatation would be that which occurs when the membranes are ruptured at the commencement of labor. It is very well known that this does not happen;" and after giving Dewees' explanation of the difficulty, proceeds: "But in place of the waters, there is the large, round, and unyielding head of the child forced down upon the lower part of the uterus, which one would suppose more efficient for the purposes of mechanical distension." According to the opinion of Dewees, and not his own, as we may fairly infer. Again, at page 50, "If the uterus exerted its full power upon the undilated os uteri, and if the unyielding head of the child were driven forcibly against it, the almost certain consequence would be, that the irritation would excite increased resistance, and ultimately terminate in inflammation of the mouth of the uterus. To obviate such an effect, nature interposes a *fluid medinm* between the power and the resistance. The liquor amnii contained within the membranes, occupies the cavity of the uterus, and when its parietes contract upon it, the force exerted is (as we have explained) by this means, accurately conveyed to the os uteri. When the latter dilates in the slightest degree, the fluid insinuates itself within the smallest opening, and expands it by a direct lateral pressure against its edges. The power of the uterus is thus made to act in the most favorable manner for distending its mouth."

On same page he says: "Further, so long as the tissue of the uterus intervenes, it is necessary to moderate the great power which the uterus is capable of exercising to dilate it: this is effected by the liquor amnii." Again, on page 108: "The gradual escape of the liquor amnii also gives rise to tediousness. If this takes place when the os uteri is slightly dilated, in other words, when the latter is so long exposed to the head of the child as to become irritated by it, the result is rigidity of the os uteri, etc." On page 55 we also read: "If they are ruptured when the dilatation is very slight, the suddenly increased power of the fundus, forcing the head of the child against the os tincæ, soon excites irritation, prevents its expansion, and sometimes causes inflammation. But if they are broken when the uterus is sufficiently open to allow the membranes to protrude into the vagina, and the contractions of the fundus to increase, it is probable that the dilatation will be advanced more rapidly, etc." This last quotation contains its own refutation. I have been thus copious in my citations, for the reason, that they embody the views of all authors within my knowledge, Dewees excepted.

Now, what inferences are fairly deducible from the foregoing quotations? I shall state and answer them as I proceed: Firstly, the amniotic sac is the essential agent in the production of dilatation of the os uteri, producing it "by a direct lateral pressure against its edges." The fact, that in a fair proportion of multiparæ labors, the uterine orifice opens readily and to some considerable extent, before either the head or membranes have engaged with it, is fatal to this theory. Further, it is a well known fact to those who have made careful observation, that occasionally in multiparæ labors, where, from fragility of the membranes, the liquor amnii has been evacuated early, and before the head has descended sufficiently to press upon the os; enlargement of the orifice proceeds without interruption.

There are three agencies concerned in the production of dilitation.

Fi.stly: and essentially, contraction of the cervico---fundal fibres acting upon the circle of the os uteri through their free extremities. Secondly, a body, (the amniotic sac or presenting part) over which to retract the border of the os; and, Thirdly: pressure by descent of the amniotic sac, or presenting portion of the fœtus.

Secondly: The amniotic sac is a more efficient dilating agent than the vertex, for the reason, that the force exerted by the uterine contractions is "accurately conveyed to the os uteri;" whereas the same force transmitted through the vertex. would "soon irritate" the os, and "prevent its expansion." Here we have groundless assumption, misinterpretation, of facts, and erroneous conclusions. By what law of mechanics does a yielding body like the amniotic sac, become a more efficient mechanical dilator than a solid body like the vertex? If the force of the contractions is (as is asserted) accurately conveyed to the edges of the os uteri,—the membranes being non-distensible, and therefore unable to absorb any of the force---why is the amniotic sac incapable of irritating and inflaming the os and arresting its dilatation, no matter how great the power exerted by the uterine walls? Can the vertex do more than transmit this force to the edges of the os uteri?

Again, it is assumed that the whole force of the uterine contractions is accurately conveyed by the amniotic liquor to the edges of the os uteri, as soon as the latter is opened in the "slightest degree." Now, the exact measure of force which the liquor amnii is capable of conveying to the edges of the os uteri, is determined by the force which the unsupported membranes at the orifice are capable of sustaining. Who can suppose for a moment, that those frail membranes can support the whole power generated by vigorous uterine contraction? Let us take even Prof. Murphy's own statement, when combatting the argument of Dewees, in regard to the antagonism of the circular and longitudinal fibres: "but how the membranes could resist the effect of this struggle it would be difficult to understand, when we know that the change of position, walking across the room, or other such trifling causes, are sometimes sufficient to rupture them, from the gravitation of the fluid alone, and therefore the greater force arising from the action of the fibres of the uterus against each other, must break them more frequently than what we know to be the case." Prof. Murphy therefore supposes, that antagonism between the vertical and transverse fibres, imparts to the liquor amnii, greater force, than when they contract conjointly. Such a view evinces a defective knowledge of hydrostatic laws. The simultaneous contraction of the uterine fibres, will distend the membranes at the orifice more forcibly than any partial contraction possibly can.

Further, it is assumed, that when the amniotic sac is prematurely ruptured, it is replaced by the vertex, which transmits the power of the uterine contractions to the os uteri, thereby irritating, and rendering it rigid and unyielding; and the conclusion is, that the tediousness of delivery, and apparent change in the condition of the os uteri, which usually attend this accident, are attributable to the alleged fact, that the vertex is incapable of dilating the os as efficiently as the amniotic sac. I have dwelt at length on this point, as the most important connected with our discussion; and an exposition of the mechanism and phenomena of the first stage of a primapara labor, will, I think, show wherein obstetrical writers have been led into fallacious reasoning, and false conclusions, by the oversight and misinterpretation of facts. At the commencement of labor, and before the os uteri begins to open; we find the child occupying the uterine cavity, with its pelvic extremity in contact with the fundus, and its head resting upon the pelvic brim, presenting the occipito-frontal diameter more or less modified,---the liquor amnii filling up the inequalities. As the contractions continue, the liquor amnii is displaced in that direction in which there is least resistance---the inferior segment; for it is a well known fact, that the muscular tissue diminishes in quantity, and therefore in power, from the fundus towards the orifice. Whilst the inferior segment is undergoing distension by the downward projection of the liquor amnii, the superior portion of the cavity is lessened, and the walls of the body and fundus permited thereby to apply themselves more closely upon the child. Then, as the cervix continues to dilate, and the os begins to open, further descent of the liquor amnii is allowed, so that the proportion of the uterine force transmitted to the pelvic brim, through the body and head of the child, increases step by step. As the os continues to open, and the membranes are more and more exposed to rupture from diminishing support inferiorly, the increasing contractile power of the uterus would certainly rupture them, were it not that, as the labor progresses, the proportion of force transmitted to, and expended upon the pelvic brim, through the child, is gradually increased. This is a wise provision of nature, for if the undivided power of the uterus should be exerted upon the unsupported membranes at the orifice as alleged by Prof. Murphy aud others, rupture of them, unless preternaturally strong, would be inevitable at an early period of dilatation, in a large majority of first labors. As the labor progresses, the uterine walls are applying themselves more firmly upon the child, during each contraction, and the head is being flexed, so as to bring its smallest circumference in relation to the superior strait. Now, suppose that at an early period of dilatation, and before flexion of the head has proceeded to any considerable extent, the amniotic sac is ruptured, either from fragility of the membranes, or interference on the part of the medical attendant; what results? Usually, abnormal protraction of delivery, aud injury to the child and mother proportionate to its duration. The cervical segment being relieved from distension, by the evacuation of the liquor amnii, retracts by its elasticity, and applies itself closely upon the presenting part, and does not come to be pressed upon until the head is completely flexed, and its sub-occipito-bregmatic circumference is passing through the superior strait. When the head is of due relative size, this step in the mechanism is never attained in the early period of dilatation, unless the cervical tissue is preternaturally unyielding. I therefore venture to assert that the statement of obstetrical writers,—Prof. Murphy included—that the ruptured amniotic sac is replaced by the vertex, which, by pressing upon the os uteri, irritates and renders it unyielding, thereby causing the tediousness which usually results where the liquor amnii is early evacuated; is entirely and radically erroneous. Further. it is a mere assumption to assert, that when the vertex, (covered by the soft, well lubricated scalp,) does come to press upon the os, it will irritate, and render it unyielding; for when, in the progress of labor, the occiput descends, it will dilate the orifice as favorably and as rapidly as the other unfavorable conditions will admit. Again, the theory implies that the delay will terminate with the full dilatation of the os, whereas it is a fact, that the delivery is delayed at every step throughout, by the too early evacuation of the liquor amnii. We have heretofore stated, that at an early period of dilatation, the head is but imperfectly flexed, and is not yet so adapted to the pelvic orifice as to prevent the outflow of liquor amnii, produced by the recurring uterine contractions. Complete flexion, and adaption of the sub-occipito-bregmatic circumference of the head to the pelvic brim, is by far the most tedious step in the mechanism of a primiparæ labor; usually requiring many hours for its accomplishment. The liqor amnii being thus gradually and completely expelled, the uterine parietes are permitted to close upon the unequal surface of the child. The circular fibres of the body, meeting with the least resistance, contract upon and constrict the abdominal region of the fœtus. Each succeeding contraction increases the constriction, until the compressed part becomes as resisting as the other portions. The child can only be expelled from the uterus by being made to glide upon its inner surface, and anything that militates against this process, will retard delivery proportionately. Now, as the resistance of the presenting part, conjoined with the resistance of this constriction, must be overcome by the contraction of the cervico-fundal fibres; the true cause of tediousness is at once made manifest; for the amount of power absolutely lost, is exactly equal to that which this constricted portion of the uterus is capable of exerting. And this relation of the uterine parietes will, at least, be partially maintained at every subsequent step of the labor, until the head is delivered ; thereby causing abnormal delay throughout.

Through the foregoing explanations, it will be perceived, that protraction and injury are liable to result, whenever the liquor amnii is evacuated before the head is properly adapted to the pelvic orifice, no matter what degree of dilatation may have taken place.

We have thus undertaken to show, "that one of its most important uses (the amniotic sac) remains unfulfilled until the presenting part has passed through the superior strait; and that in all natural, *uncomplicated* labors, especially primaparæ, the membranes should not be ruptured at any time."

In cases of actual inertia the practitioner must exercise his judgment, in view of the conditions present , but *rigidity of the membranes* can in no way impede the descent of the presenting part through the pelvic brim. Therefore it is my deliberate opinion, derived from careful and extended observation, that rupture of theamniotic sac, especially in primaparæ cases, before the head is properly adapted to the pelvic orifice ; is injudicious, and generally injurious, and sometimes fatal,—especially to the child, in its results. For, the altered condition of the uterine sinuses and the placenta, consequent upon prolonged and undue retraction of the uterine parietes, in conjunction with the violent pressure to which the infant is subjected ; are well calculated seriously to impair the child's viability, even though it may be born alive.

There are other and important objections to rupturing the membranes, even when the os uteri is well dilated. Who knows at what period of a labor eclampsia may occur ? Version may be desirable, but difficult and dangerous if the waters have been long evacuated. The same may be said in regard to rupture of the uterus. So also, if hæmorrhage ; the first expedient being rupture of the membranes, the next, version.

In conclusion : as it is difficult to estimate in a given case of labor, to what extent its bad results may be fairly attributable to improper management, we may feel disposed to rest satisfied with our preconceived ideas and modes of practice. Let us, however, be reminded of the statistical fact,—established by the observations of Burns, Collins, Nagaele, Simpson and others ; *that the liability to injury and death, upon the part of the mother and child, increases proportionately with the duration of labor.*— *Western Lancet.*

